# A Polydimethylsiloxane (PDMS) Waveguide Sensor that Mimics a Neuromast to Measure Fluid Flow Velocity †

**DOI:** 10.3390/s19040925

**Published:** 2019-02-22

**Authors:** Bianca Wiesmayr, Markus Höglinger, Michael Krieger, Philip Lindner, Werner Baumgartner, Anna T. Stadler

**Affiliations:** 1 Institute of Biomedical Mechatronics, Johannes Kepler University Linz, 4040 Linz, Austria; markus.hoeglinger@oeh.jku.at (M.H.); werner.baumgartner@jku.at (W.B.); anna.stadler@jku.at (A.T.S.); 2 Institute of Fluid Mechanics and Heat Transfer, Johannes Kepler University Linz, 4040 Linz, Austria; michael.krieger@jku.at; 3 Institute of Semiconductor and Solid State Physics, Johannes Kepler University Linz, 4040 Linz, Austria; philip.lindner@jku.at; 4 Center for Surface and Nanoanalytics, Johannes Kepler University Linz, 4040 Linz, Austria

**Keywords:** biomimetic flow sensor, PDMS waveguide, optical detection principle

## Abstract

Accurate flow measurement is a ubiquitous task in fields such as industry, medical technology, or chemistry; it remains however challenging due to small measurement ranges or erosive flows. Inspiration for possible measurement methods can come from nature, for example from the lateral line organ of fish, which is comprised of hair cells embedded in a gelatinous cupula. When the cupula is deflected by water movement, the hair cells generate neural signals from which the fish gains an accurate representation of its environment. We built a flow sensor mimicking a hair cell, but coupled it with an optical detection method. Light is coupled into a PDMS waveguide that consists of a core and a cladding with a low refractive index contrast to ensure high bending sensitivity. Fluid flow bends the waveguide; this leads to a measurable light loss. The design of our sensory system allows flow measurement in opaque and corrosive fluids while keeping production costs low. To prove the measurement concept, we evaluated the light loss while (a) reproducibly bending the fiber with masses, and (b) exposing the fiber to air flow. The results demonstrate the applicability of an optical fiber as a flow sensor.

## 1. Introduction

In biomimetics, natural structures are first analyzed in depth and then, an abstract description is developed to translate the biological concepts to the target discipline. Plants, animals, and microbes are adapted to their respective ecological niches. The strategies that can be observed in nature today have prevailed for a long time and have therefore been tested thoroughly. Understanding these strategies and applying them in problem-solving processes may help to develop novel technologies and to optimize existing ones [1,2].

A model of flow sensing, for example, can be found underwater. All fish and amphibians living permanently in water have a lateral line organ, which detects water disturbances and thus provides information that is essential to survival. Hence, the organ contributes to the formation of schools of fish, to sensing of the trails of prey or predators, and to communication within species [2,3].

**Figure 1:**
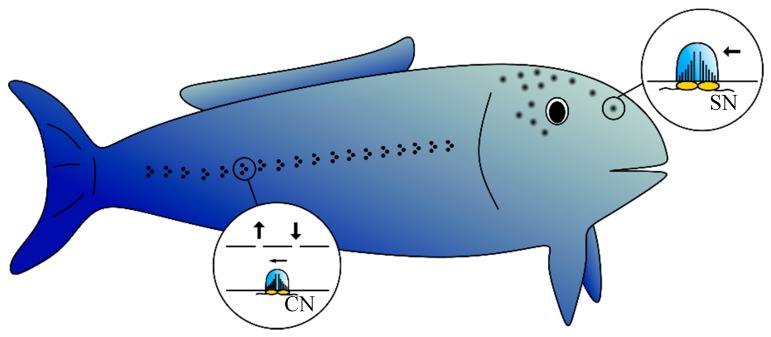
Neuromasts are distributed across the body of the fish, while the exact placement varies between species. Superficial neuromasts (SN) are located on the surface of the skin, while canal neuromasts (CN) are found in a canal beneath the skin that is connected to the environment via pores.

The fish’s lateral line (see Figure 1 for an example structure) consists of hundreds of neuromasts distributed across its head and body [3]. A neuromast is comprised of hair cells embedded in a gelatinous cupula. Each hair cell has a hair bundle on the outer cell surface that consists of a kinocilium and up to 150 shorter stereovilli [2,4]. Fluid flow deflects the cupula and thus bends the hair, and the cell emits a neural signal. The neuromasts in the lateral line can be classified into two types: superficial (SNs) and canal (CNs) neuromasts. While SNs occur on the surface of the skin, CNs are located between pairs of pores in a canal under the skin. SNs measure the fluid flow velocity of the bulk water flow, and CNs record pressure differences between the canal pores [5]. 

Numerous artificial flow sensors that mimic neuromasts have been developed. The biomimetic sensors hitherto created are based on various principles such as hot-wire anemometry [6], capacitance measurement [7], piezoelectricity [8], and ionic polymer-metal composite sensing [9]. Furthermore, a polydimethylsiloxane (PDMS) sensor using optical detection and implemented as a micro-electromechanical system (MEMS) was presented [10,11]. 

In several studies [6,8,9,12,13,14], biomimetic flow sensors were combined to an artificial lateral line, which may be useful, for instance, in autonomous underwater navigation [15]. Individual sensors have been used for gas-flow sensing [10], underwater flow sensing [7,8,9,11], and tap-water measurement [11].

The purpose of this study was to investigate a novel, low-cost and robust fluid-flow sensor. Addressing a possible limitation of the measurement setup previously described by a German research group [10,11], the design of our sensory system also allows flow measurements in opaque and erosive fluids. 

Most biomimetic sensors hitherto created are fabricated as MEMSs. We focused on increasing the robustness of the sensors and, therefore, our sensor system is optimized for flow measurements in tubes with a diameter of 30 mm to 50 mm. The PDMS sensor fiber can be removed from the setup and therefore autoclaving is feasible, which is important for applications in medical technology. The fabrication method and selection of materials aim at fabricating a robust but nevertheless low-cost sensor.

After analyzing the natural structures, we developed an analogy for use in our flow sensor. Deflection of a neuromast is mimicked by an elastic optical waveguide that is exposed to fluid flow. It comprises a core and a surrounding cladding. The core material has a higher refractive index than the cladding, which results in a waveguiding effect due to total internal reflection. Optical losses are produced when the fiber is bent. Our waveguide is optimized for high bending losses, as we selected two materials with similar refractive indices. 

In a previous study, a similar sensor setup was used to measure water flow [16]. Furthermore, the requirements of a sensor for application in spirometry were outlined [17]. The primary objectives of this study were (a) the design of an optical fiber with high bending sensitivity, (b) the design of a measurement setup, and (c) the experimental proof of concept.

## 2. Materials and Methods

**Figure 2:**
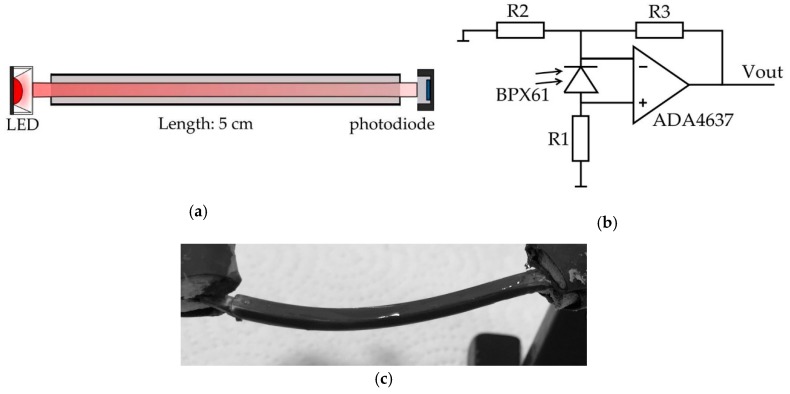
Measuring the deflection of the waveguide: (**a**) Schematic of the optical sensory system that comprises an LED, an optical waveguide and a photodiode [17]. (**b**) Photodiode amplifier [18]. (**c**) Fabricated PDMS waveguide consisting of three layers (core, cladding, sealing).

The LED is driven by a constant current source of 100 mA and the op-amp has a maximum symmetric supply of 15 V. The operation principle is shown in Figure 2a: Red light is coupled into the waveguide by an LED (XP-E2, Cree, Durham, North Carolina, USA) after being focused by a lens (18°, Carclo Optics, Aylesbury, UK). At the other end of the waveguide, the remaining light is detected by a photodiode (BPX61, Osram, Munich, Germany). The fiber has a length of 5 cm, and a square cross-section of 3 × 3 mm^2^. 

### 2.1. PDMS Waveguide

The fabrication process of the optical waveguide has been described and illustrated by Stadler et al. [17]. The core is made of Sylgard 184 elastomer cast in an aluminum mold and cured at 145 °C for 20 min. Sylgard 184 has a slightly higher refractive index than the cladding material RTV 615. After integration of the core into the mold for the cladding, the material is poured in and cured at 145 °C for 20 min. 

Schneider et al. [19] analyzed the optical properties of various PDMS materials, showing that Sylgard 184 has an absorption minimum around 620 nm. Therefore, red light was chosen for the optical path. At a wavelength of 625 nm, the refractive indices amounted to 1.4299 (Sylgard 184) and 1.4286 (RTV 615) [19]. A small refractive index contrast results in the high bending sensitivity of the waveguide [20].

An additional sealing layer (RTV 3145) was applied to (a) protect the fiber against erosion, (b) shield the waveguide from ambient light, and (c) absorb leaky rays and hence avoid that they tunnel back into the fiber core [20].

The sealing silicone RTV 3145 is highly viscous and was therefore diluted with toluene before applying it onto the waveguide with a brush.

### 2.2. Photodiode and Photodiode Amplifier

The photodiode has a high sensitivity of 0.62 A/W, allowing detection of low light levels. Although the device has a large active area of 7 mm^2^, the capacitance amounts to only 72 pF [21]. Since the signal is amplified by a photodiode amplifier, the output voltage depends linearly on the photocurrent. As proposed by Horowitz and Hill [18], the photodiode is placed between the inputs of the op-amp (ADA4637-1ARZ, Analog Devices, Norwood, Massachusetts, USA) for nearly zero bias voltage and noise reduction. The design was optimized for low noise and for an output voltage that is largely independent of temperature in order to increase accuracy. Furthermore, interferences had to be reduced by incorporating the setup into an aluminum shielding cage and by using shielded cables. 

The selected op-amp has a low input bias current of 5 pA as well as a low offset voltage of 200 µV. The input bias current leads to an absolute measurement error as it influences the photocurrent, while the offset voltage constitutes the bias voltage of the photodiode. The temperature drift of most parameters of the op-amp is insignificant. In addition, a large bandwidth and slew rate allow the recording of fast changes of the light loss [22].

The circuit was simulated in LTSpice to adapt the gain of the setup. The desired output voltage range was 0 V to 10 V for an expected photocurrent of up to 500 nA. A 400 kΩ resistor (R1; see Figure 2b) was chosen to convert the photocurrent into a voltage. Resistors R2 and R3 of 1 and 60 kΩ respectively, define the amplification. The circuitry was fabricated as a printed circuit board (PCB) with high-precision surface-mount devices (SMD). All selected resistors had a low temperature drift of 5 ppm/K (R2, R3) or 10 ppm/K (R1). In the layout, R1 and R3 were split into two antiparallel resistors with half the resistance to further reduce interferences. The photodiode (through-hole technology) was placed directly on the PCB. For increased stability, a capacitor (70 pF) was added parallel to R4. Decoupling capacitors (100 nF) were placed close to the supply pins of the op-amp.

### 2.3. Measurement Setup

#### 2.3.1. Setup 1: Bending Masses Simulate Fiber Deflection

**Figure 3:**
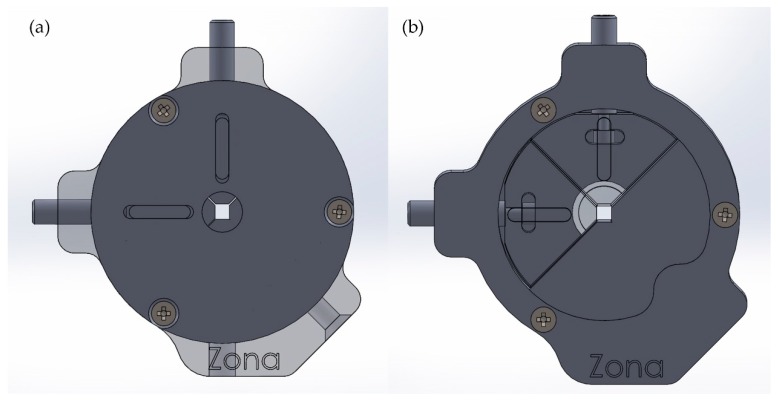
Fiber clamps that are specifically designed for the application. For our measurements, it is essential that the elastic fiber is reproducibly clamped without being twisted or squeezed. (**a**) Closed view of one fiber clamp, and (**b**) open view to visualize the clamping mechanism.

Measurement Setup 1 is shown in our previous work [17]. The fiber was fixed with two clamps (Figure 3) that were particularly designed to fix the fiber without squeezing and to prevent fiber twisting. The fixation method ensures reproducible results, even when the fiber is replaced.

The output voltage was recorded using a data acquisition device (DAQ, NI USB-6228, National Instruments, Austin, Texas, USA) connected to a laptop via USB. A LabVIEW script (LabVIEW 2014, National Instruments, Austin, Texas, USA) evaluated and saved the data. The PDMS waveguide was tested for masses bending the sensor to simulate deflection of the waveguide in fluids. For each measurement, the reference voltage V_0_ of the unbent waveguide was recorded. The output voltage decreased when a mass stressed the waveguide; this light loss ΔV corresponded to the bending mass. 

Three cycles consisting of 100 individual measurements each were performed. For the first 50 data points in each cycle, the bending mass was increased from 0.1 g to 5 g, while for the others, it was reduced from 5 g to 0.1 g. The masses were implemented as lead-filled micro test tubes between 0.1 g and 5 g with a step size of 0.1 g. They were attached to the center of the fiber with a nylon thread. Data were recorded for 5 s with a sampling frequency of f = 4 kHz, and the mean value was calculated after discarding 10% outliers to reduce oscillations.

#### 2.3.2. Setup 2: Fluid Flow Measurements

**Figure 4:**
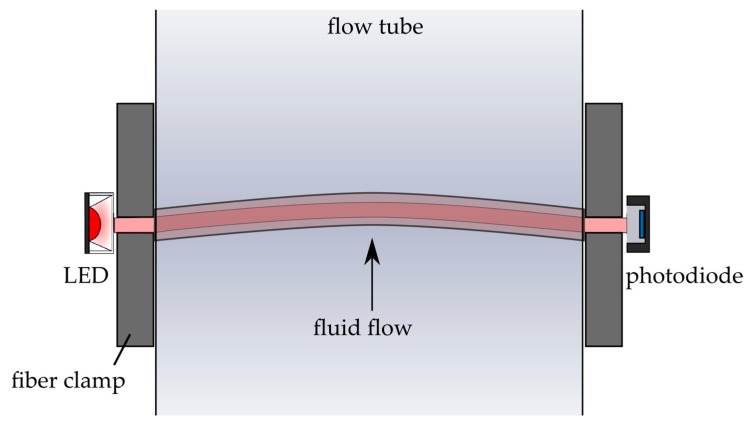
Schematic description of the sensor and the sensor mechanism in the presence of fluid flow. The fiber is integrated into a flow tube while fixed on each side with fiber clamps. Fluid flow bends the waveguide and the light intensity at the output decreases.

**Figure 5:**
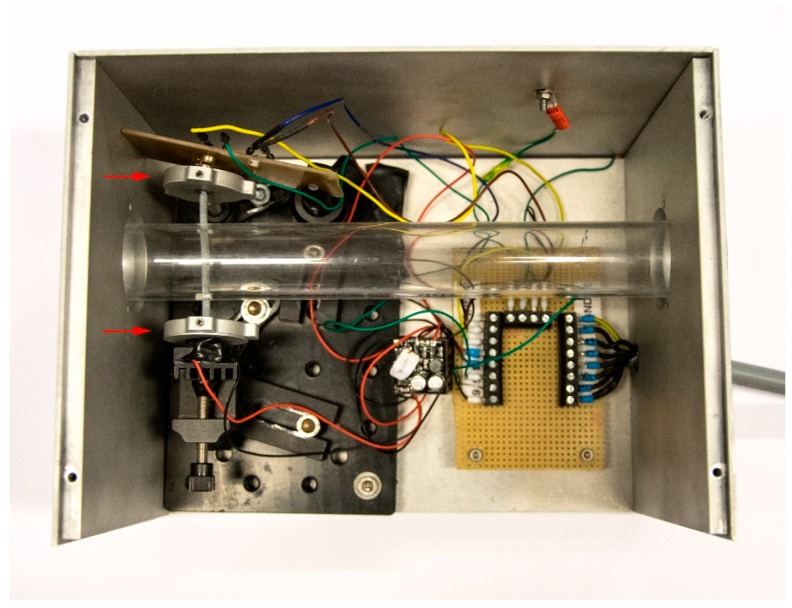
Setup for measurements in air flow. The fiber was integrated into a transparent flow tube (Ø 30 mm) with fiber clamps (red arrows) on each side to ensure a reproducible fixation. The setup is incorporated into an aluminum cage to shield the electronics against interferences. A ventilator on one side of the tube creates the air flow.

After a successful experimental proof of concept, Setup 1 was adapted to test the sensor in fluid flow (see Figure 4 and Figure 5). The waveguide was put into an acrylic glass (PMMA) pipe with an inner diameter of 30 mm. Therefore, the fiber length was changed accordingly. 

The air flow was generated by an axial fan (Sanyo Denki San Ace 36, RS components, Corby, UK), that was connected to the pipe via a pipe adapter. The output voltage was recorded by means of an oscilloscope (RIGOL DS1104Z Plus, deg-Messtechnik, Tulln, Austria), which was connected to a laptop via USB. The Virtual Instrument Software Architecture (VISA, National Instruments, Austin, Texas, USA) was used to communicate with the oscilloscope. The data were saved and evaluated with MATLAB (R2015b, Mathworks, Natick, Massachusetts, USA). As the flow velocity depends on the supply voltage of the fan, this voltage was varied between 4.2 V and 13 V. 

Three cycles of approximately 30 individual measurements were conducted. In each cycle, the voltage was first increased from 4.2 V to 13 V and, subsequently, decreased from 13 V to 4.2 V again.

A hot-wire anemometer (Testo 405i, RS components, Corby, UK) was applied to measure the air flow velocity generated in the setup. 

## 3. Results and Discussion

### 3.1. Bending Induced by Masses

**Figure 6:**
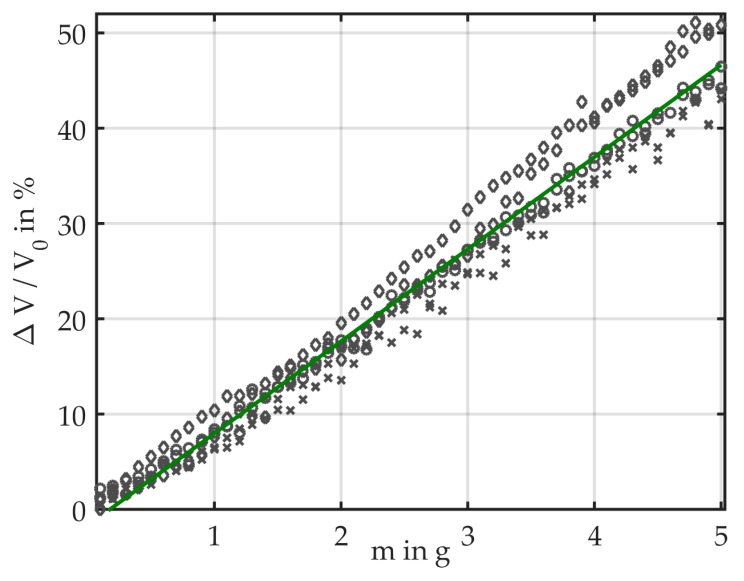
Measurement results showing that the light loss relative to the reference voltage depends linearly on the bending mass. Results of six measurements with masses from 0.1 g to 5 g (step size 0.1 g) including linear approximation are plotted.

**Figure 7:**
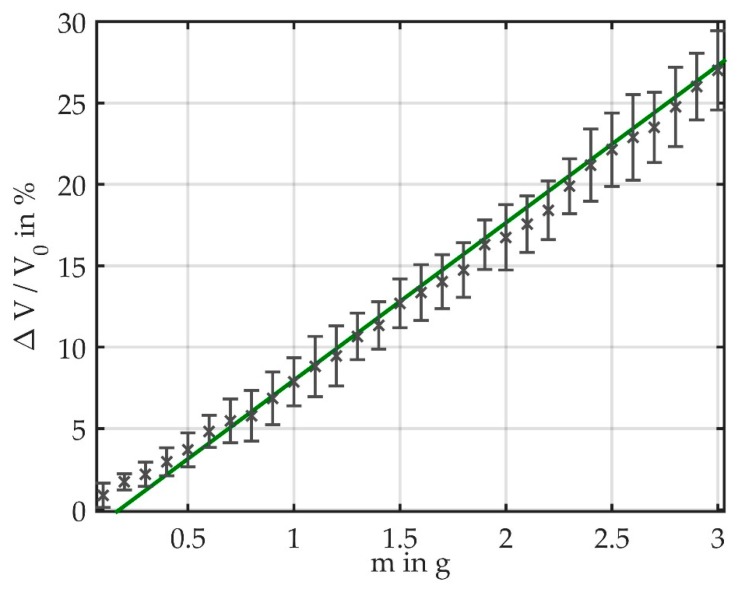
Measurement results showing that the light loss relative to the reference voltage depends linearly on the bending mass. The average measurement results including error bars for bending masses of 0 g to 3 g are shown.

For each bending mass, we calculated the light loss relative to the reference voltage V_0_ of the unbent fiber. We obtained the best results when using V_0_ measured at the start of a measurement cycle. Figure 6 shows the data of six measurement cycles and illustrates that the sensor shows a linear behavior toward bending with masses. The linear fit has a slope of 9.67, which corresponds to the sensitivity of the sensor. The average light loss was calculated for each bending mass, which is illustrated together with the standard deviation in Figure 7. It provides a more detailed view of the measurement data and comprises bending masses between 0 g and 3 g. The measurement results are plotted as relative light loss over the attached mass. The results show that the light loss is linearly dependent on the induced bending. At a mass of 5 g, the mean light loss is 46.7%; therefore, the average loss for Δm = 0.1 g amounts to approximately 1%. The standard deviation increases with the attached mass.

The standard deviation ranges from 0.75% at 0.1 g to 4.36% at 4.9 g. It increases for higher masses because the attached masses visibly twist the fiber. This random measurement error is inherent to the measurement setup, and is expected to be less significant when operated in continuous air flow. This also implies that the accuracy of the sensor depends on the range of the masses. To calculate the accuracy we therefore included only the light loss for bending masses ≤3 g (Figure 7). At 1 g, the light loss was 6.85% ± 1.62%; at 2 g, it amounted to 16.74% ± 2%; and at 3 g, 27% ± 2.43%. For the measurement range up to 3 g, the standard deviation does not exceed 2.63% (occurring at 2.6 g). 

The advantage of this setup is the high reproducibility of the measurements. It is a feasible proof-of-concept method to show the behavior of the fiber over a range of bending forces. 

### 3.2. Exposing Fiber to Air Flow

**Figure 8:**
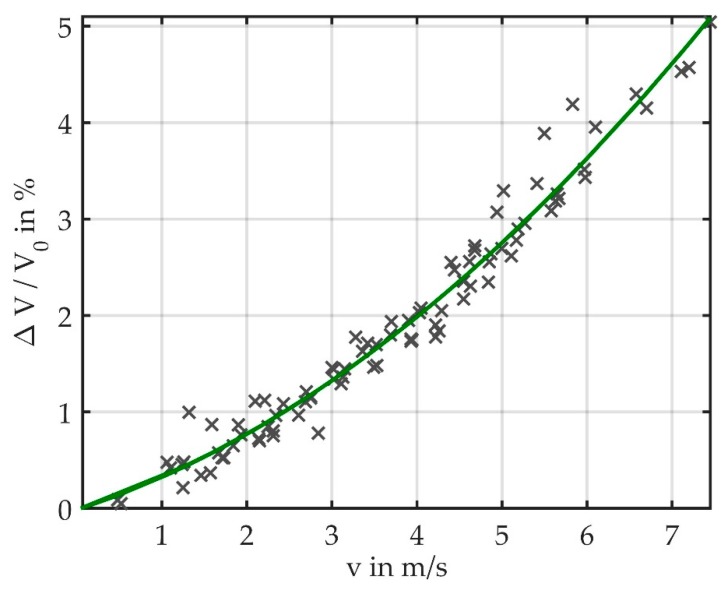
Measurement results showing that the light loss depends quadratically on the flow velocity. Results of three measurements with flow velocities from 0.48 m/s to 7.5 m/s are marked as well as the quadratic fit.

Results of the measurements in air flow are illustrated in Figure 8. By varying the supply voltage of the axial fan, air velocities between 0.48 m/s and 7.45 m/s could be reached. The maximum flow velocity was limited by the supply range of the axial fan. The light loss in this velocity range varied between 0.05% and 5.04%. As the waveguide is directly connected to the LED and the photodiode, measurements in opaque environments are feasible. 

Although the same set of supply voltages was applied in each measurement, the flow velocities measured by means of the testo constant temperature anemometer (CTA) scattered (see Figure 8) due to the significant measurement error of the testo device. Between 0.1 m/s and 2 m/s, the measurement error amounts to 0.1 m/s + 5% of the result; between 2 m/s and 15 m/s it amounts to 0.3 m/s + 5% of the result. We thus could not calculate confidence intervals, the results however show that the light loss depends quadratically on the flow velocity. 

The setup is designed so that it could also be used for lung function testing. The studied flow range corresponds to the flow range that is observed in the human respiration cycles. As the fiber can be autoclaved the required hygiene standards for this application may be met.

### 3.3. Comparison of the Measurement Setups

When comparing the results of the two setups (Figure 6 and Figure 8), it is important to consider the different measurement ranges. While in Setup 1 the light loss reaches up to 46.7%, in Setup 2 it varies between 0 and 5.04%. It is obvious that, due to the more consistent bending, the measurement results in continuous air flow can be better reproduced than in Setup 1, where the masses induce a fiber twist that leads to a random measurement error. 

### 3.4. Fluid Flow around an Elastic Fiber

Even though the flow measurement results clearly indicate a quadratic relationship of light loss and flow velocity, scatter in the data is significant. This can at least partly be attributed to measurement uncertainties of the setup, but might also result from the fluid flow around the fiber. The following discussion assumes that an elastic fiber is placed in a straight tube of circular cross section (diameter D_T_ = 30 mm, blockage ratio 3.2%). The mean air velocity in the tube may vary between 0 and 7.5 m/s. The Reynolds number Re based on the tube diameter ranges from 0 to 14,700, encompassing the laminar and turbulent flow domain. The Reynolds number Re_L_ based on the fiber side-length of 3 mm can vary between 0 and 1,470. Several different flow regimes can occur for a bluff body in cross-flow in this range of Reynolds numbers. Especially the aerodynamics of a circular cylinder in uniform cross-flow has been studied extensively and is discussed in numerous fluid-mechanic text books (e.g., [23]). For Reynolds numbers above approximately 40, alternating vortex shedding occurs, which leads to oscillations of the drag and lift coefficients. The vortex-shedding frequency f is usually expressed in terms of the Strouhal number St = f L/u, with u as the cross-flow velocity. In the case of a circular cylinder the length scale L stands for the cylinder diameter, and in the case of a square cylinder for its side-length B. A detailed review of the different flow regimes of the circular cylinder wake is given in [24]. Even though aspect ratio, blockage, surface roughness, turbulence, end design, and cylinder vibrations all effect vortex shedding, similar trends as those described in [24] can be observed for all cylinder wakes. For Reynolds numbers ranging up to 3000, Williamson [24] distinguishes between five regimes. The laminar steady regime (Re_L_ < 49) is characterized by symmetric vortices forming behind the cylinder. Laminar vortex shedding (Kármán vortex street) can be observed for Reynolds number up to around 190. This is followed by a transition regime to three-dimensional vortex formation (Re_L_ < 260), where two different modes of instability can occur depending on the Reynolds number. The three-dimensional vortex formation becomes increasingly disorderly until a fully turbulent wake forms (Re_L_ > 1000). The flow around square cylinders differs from that of circular cylinders, since flow separation is—with the exception of very low Reynolds numbers—fixed at the leading edge corners. Nonetheless it exhibits similar behavior concerning wake formation with slightly different transition Reynolds numbers between the flow regimes. Measurements and numerical simulations for square cylinders (e.g., [25,26,27]) show that the Strouhal number ranges from 0.1 to 0.17, with peak values at Re_L_ = 200 and that temporal variations of lift and drag coefficients vary significantly over different flow regimes. Vortex-induced vibrations of square cylinders strongly depend on structural mechanics (spring and damping coefficients) as well as the ratio of replaced mass and cylinder mass [28]. All the aspects outlined above, as well as the tube flow profile, must be accounted for in order to study the flow-induced deformation of an elastic fiber. The complexity of the flow situation will necessitate further research and certainly careful calibration in order to achieve the required accuracy for an optical flowmeter.

## 4. Conclusions

A fish’s lateral line measures fluid flow velocity by sensing the deflection of neuromasts. We abstracted the biological model of the superficial neuromast to design an optical flow sensor. A PDMS waveguide is used as an optical waveguide with high sensitivity to bending losses. The biomimetic sensor principle has several advantages, as it allows measurement in corrosive and opaque fluids, and autoclaving of the fiber. This makes it a promising fluid flow sensor in various fields, such as medical engineering and chemistry.
